# S14-4: Designing a physical activity pathway in healthcare model for Irish Health Services

**DOI:** 10.1093/eurpub/ckae114.263

**Published:** 2024-09-26

**Authors:** Sarah O’Brien, Blathin Casey, Eimear Cotter

**Affiliations:** Health Service Executive Ireland; Health Service Executive Ireland; Health Service Executive Ireland

## Abstract

**Background:**

There is strong evidence for health benefits of regular physical activity (PA) for preventing non-communicable diseases and reducing risk factors as well as improving mental, physical, and emotional health for those living with chronic diseases. There is not a consistent, coherent mechanism to connect patients in primary care with supports and services to be more physically active currently within the Irish public health service.

**Purpose:**

To design a physical activity pathway, and identify the supporting infrastructure required to enable implementation across the Irish public health system, with a particular focus on primary care.

**Methods:**

Using HSE Change Management Guide, the HSE Healthy Eating Active Living Programme commenced a project to co-design a pathway and identify essential infrastructure to support implementation of the pathway across the Irish public health system. Stakeholders engaged in the co-design process include health professionals, health service planners and managers, Local Sports Partnerships, Sport Ireland as well as the public who are potential service users.

**Results:**

A range of methods including interviews, focus groups and workshops were used to gather insights from stakeholders and potential service users to inform the service design. The COM-B model was used as an underpinning behaviour change theoretical framework to analyse the insights including the use of APEASE criteria to prioritise components for the service design.

**Conclusions:**

Establishing a physical activity pathway in healthcare model in a public health service is a complex process. Using a structured change management process, with a strong focus on stakeholder engagement to facilitate co-design may increase success of implementation.

**Practical implications:**

This work will result in a coherent, consistent, resourced process to connect service users in the Irish public health service with appropriate community based, publicly funded physical activity opportunities.

**Funding:**

The Health Services Executive, Ireland

